# Jejuno-jejunal intussusception secondary to feeding jejunostomy tube: a case report

**DOI:** 10.11604/pamj.2024.49.12.44774

**Published:** 2024-09-09

**Authors:** Youssef Motia, Dounya Douah, Smail Sourni, Said Bassel, Lamribah Mohamed, Youssef Harrouni, Hamza Haddani, Ayoub Madani, Mohamed Ouazni, Mehdi Soufi

**Affiliations:** 1 Department of General Surgery, Souss Massa University Hospital Center, Agadir, Morocco,; 2 Faculty of Medicine and Pharmacy, Ibn Zohr University, Agadir, Morocco

**Keywords:** Acute intestinal intussusception, jejunostomy tube, intestinal obstruction, surgery, case report

## Abstract

Feeding jejunostomy is a simple and common procedure used to provide enteral nutrition. Acute intestinal intussusception on a jejunostomy tube is a rare complication that can have catastrophic consequences and often requires urgent surgical intervention. We report the case of a 45-year-old female patient with a stenosing hypopharyngeal tumor leading to complete aphagia. Due to the severe deterioration of her general condition, the patient underwent surgery, and a Witzel-type feeding jejunostomy was performed. The patient's postoperative course was notable for the development of intussusception around the jejunostomy tube two months later, which required surgical intervention. The recovery was uneventful. Early diagnosis is crucial to improve the prognosis of this particular form of acute intestinal intussusception. Treatment is almost exclusively surgical.

## Introduction

Acute intestinal intussusception of the small bowel is an extremely rare complication in adults, characterized by the telescoping or invagination of a proximal segment into the lumen of an adjacent segment. It often occurs secondary to infectious or organic lesions [1]. However, acute intestinal intussusception secondary to a jejunostomy tube is a particularly rare abdominal emergency, accounting for 2.5% to 18% of acute intestinal intussusception cases [1]. Clinically, it presents as high intestinal obstruction. Diagnosis can be made either preoperatively using imaging techniques or intraoperatively. Treatment is most often surgical [1].

Here, we report a rare case of acute intestinal intussusception in an adult, complicating a jejunostomy, with non-specific clinical signs, which required surgical intervention. This case highlights the critical need for prompt recognition and management of intussusception in patients with jejunostomy to prevent serious and potentially life-threatening complications.

## Patient and observation

**Patient information:** a 45-year-old female patient with hypopharyngeal cancer presented with total aphagia and a deteriorated general condition. She underwent surgery, during which a Witzel-type feeding jejunostomy was placed due to severe malnutrition. The immediate postoperative course was uneventful, and the patient was discharged on the third postoperative day. Two months later, she was admitted to our emergency department with signs of intestinal obstruction.

**Clinical finding:** clinical examination revealed a weakened and dehydrated patient with a slightly distended and tender abdomen, along with reflux of small bowel contents through the jejunostomy opening.

**Timeline of current episode:** this is illustrated in Table 1.

**Table 1:** timeline of the current episode

Timeframe	Event
08/02/2024	Witzel-type feeding jejunostomy
11/02/2024	The immediate postoperative course was uneventful
	Patient was discharged
06/04/2024	Admission to the emergency department with signs of intestinal obstruction
	Clinical examination + plain abdominal X-ray
	Surgery
08/04/2024	Resumption of bowel function
09/04/2024	Smooth recovery (no complications)
	The patient expressed satisfaction with the treatment
	Patient was discharged

**Diagnostic assessment:** a plain abdominal X-ray showed dilated small bowel loops and multiple air-fluid levels, which are highly suggestive of small bowel obstruction. Abdominal ultrasonography and computed tomography (CT) were not accessible in the emergency setting.

**Figure 1:**
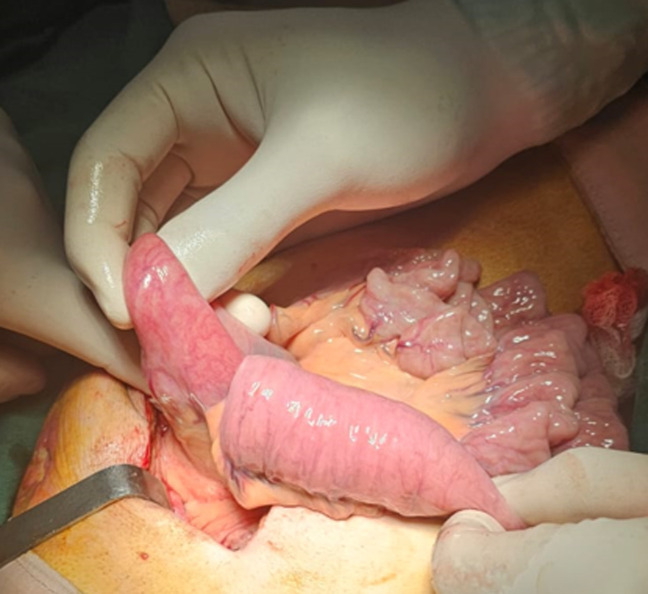
intraoperative appearance of intestinal intussusception

**Diagnosis:** given this presentation, urgent surgical intervention was indicated. Intraoperatively, an anterograde proximal intussusception was discovered 40 cm from the Treitz ligament, extending over 10 cm, with the feeding jejunostomy tube inside. The bowel was nonischemic (Figure 1).

**Figure 2:**
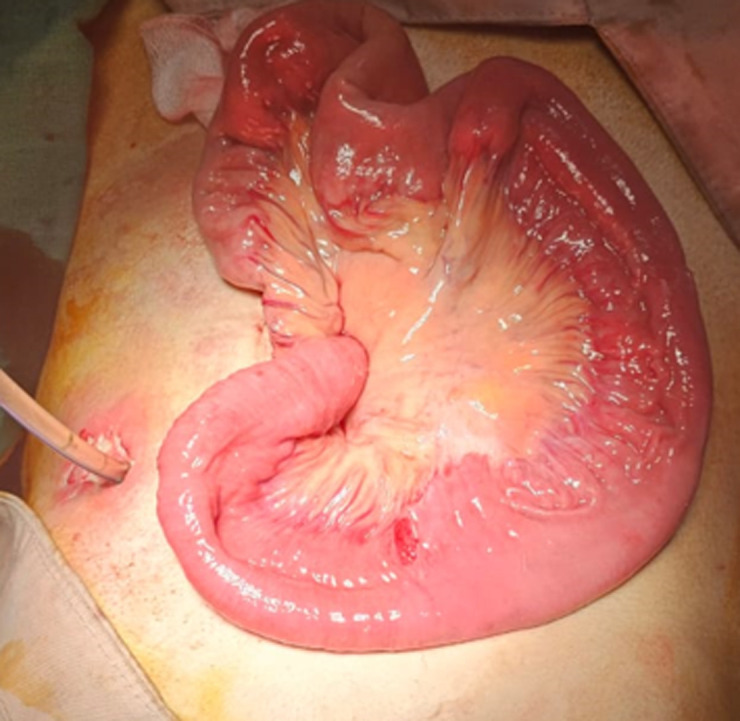
appearance after reduction of intussusception

**Therapeutic intervention:** the surgical procedure included manual reduction of the intussusception (Figure 2) and placement of a new feeding jejunostomy tube.

**Follow-up and outcome of interventions:** the postoperative course was uncomplicated, with the resumption of bowel function on the second day.

**Patient perspective:** our patient was very satisfied with the provided treatment. She felt reassured and well-informed, which strengthened her trust in our team. She was grateful for the quick intervention and effective relief of her symptoms.

**Informed consent:** informed consent was obtained from the patient.

## Discussion

Acute intestinal intussusception related to a jejunostomy tube is an uncommon abdominal emergency, representing under 1% of all small bowel obstruction cases and 5% of intussusception cases overall. Common complications associated with feeding jejunostomy include mechanical obstruction or infections [1]. Intussusception can be either antegrade or retrograde. The average age of onset is 50 years, with a male-to-female ratio of 1 [2].

From a pathophysiological perspective, acute intestinal intussusception may arise from disrupted peristalsis caused by material within the intestinal lumen or damage to the intestinal wall [3]. A literature review suggests that acute intestinal intussusception could result from retrograde peristalsis of the jejunum during vomiting episodes, or from the stent-like effect of the feeding tube's infusion force. Additionally, many patients requiring tube feeding are thin with minimal adipose tissue (omentum, mesentery) in the abdominal cavity, allowing greater mobility of the small intestine and potentially increasing the risk of intussusception [4]. Other risk factors include digestive tract spasms, abnormal peristalsis, postoperative adhesions, stimulation due to surgical manipulation, and postoperative inflammation [5]. A study by Carucci *et al*. reported complications in 14% of the 280 patients with feeding jejunostomy, including obstruction, small bowel stenosis, and intra-abdominal collections. Rarer complications, such as hematomas and small bowel intussusception (1%) at the jejunostomy site, have also been documented [6].

Intestinal intussusception is rarely encountered in adults [7]. Its diagnosis can be complex due to non-specific clinical symptoms. Suggestive signs include abdominal pain, palpable abdominal mass, and bloody stools [8]. Intussusception can also cause small bowel obstruction with reflux of digestive fluid through the feeding jejunostomy stoma, as observed in our patient. Preoperative diagnosis is often challenging, with suspicion rates ranging from 14% to 75% according to literature data [9]. While ultrasound may reveal «target» or «sandwich-like images», its effectiveness can be limited by obesity and abdominal distension. Abdominal CT remains the most sensitive method for confirming the diagnosis, providing critical information on the length and type of intussusception [5]. Additionally, CT can detect hyperdense material within the intussusception, which is highly indicative of the jejunostomy tube [5]. In the case of our patient, performing an emergency abdominal CT was not feasible without delaying treatment. Consequently, we proceeded with surgical exploration, which confirmed the diagnosis.

Regarding therapeutic management, it primarily relies on surgery. Liao G-S *et al*. recommended surgery for all cases showing signs of obstruction [10]. There is controversy regarding the necessity of reducing the intussusception before resection. Due to the high incidence of associated cancer (1% to 40%), small bowel intussusception should be reduced only if the benign nature is confirmed preoperatively [10]. Furthermore, if resection risks causing short bowel syndrome, it is preferable to attempt reduction to preserve intestinal length [10]. In our patient, we opted for manual reduction without resection due to the absence of necrosis during surgical exploration. Following certain nutritional guidelines described by the American Society of Nutrition is recommended to ensure proper functioning of enteral nutrition via jejunostomy [2]. This preventive strategy includes initiating enteral feeding after resumption of bowel function, increasing the number of lateral holes in the jejunostomy tube to reduce pressure, starting feeding at a rate of 10-20 ml/h, and performing wide suturing of the jejunum [2].

## Conclusion

Acute intestinal intussusception secondary to jejunostomy tubes is a rare complication and a surgical emergency requiring early diagnosis and treatment. This condition should be suspected when a patient with feeding jejunostomy develops upper gastrointestinal obstructive symptoms, especially when they are improved by nasogastric tube drainage. The diagnosis is confirmed through ultrasonography or CT, although surgical exploration may sometimes be necessary.
